# Machine Learning-Based Predictive Maintenance at Smart Ports Using IoT Sensor Data

**DOI:** 10.3390/s25133923

**Published:** 2025-06-24

**Authors:** Sheraz Aslam, Alejandro Navarro, Andreas Aristotelous, Eduardo Garro Crevillen, Alvaro Martınez-Romero, Álvaro Martínez-Ceballos, Alessandro Cassera, Kyriacos Orphanides, Herodotos Herodotou, Michalis P. Michaelides

**Affiliations:** 1 Department of Electrical Engineering, Computer Engineering, and Informatics, Cyprus University of Technology, Limassol 3036, Cyprus; am.aristotelous@edu.cut.ac.cy; 2 Prodevelop S.L, 46001 Valencia, Spain; anavarro@prodevelop.es (A.N.); egarro@prodevelop.es (E.G.C.); amromero@prodevelop.es (A.M.-R.); aceballos@prodevelop.es (Á.M.-C.); 3 Eurogate Container Terminal Limassol Ltd., Limassol 3045, Cyprus; alessandro.cassera@eurogate-limassol.com (A.C.); kyriacos.orphanides@eurogate-limassol.com (K.O.)

**Keywords:** predictive maintenance, smart ports, machine learning, IoT

## Abstract

Maritime transportation plays a critical role in global containerized cargo logistics, with seaports serving as key nodes in this system. Ports are responsible for container loading and unloading, along with inspection, storage, and timely delivery to the destination, all of which heavily depend on the performance of the container handling equipment (CHE). Inefficient maintenance strategies and unplanned maintenance of the port equipment can lead to operational disruptions, including unexpected delays and long waiting times in the supply chain. Therefore, the maritime industry must adopt intelligent maintenance strategies at the port to optimize operational efficiency and resource utilization. Towards this end, this study presents a machine learning (ML)-based approach for predicting faults in CHE to improve equipment reliability and overall port performance. Firstly, a statistical model was developed to check the status and health of the hydraulic system, as it is crucial for the operation of the machines. Then, several ML models were developed, including artificial neural networks (ANNs), decision trees (DTs), random forest (RF), Extreme Gradient Boosting (XGBoost), and Gaussian Naive Bayes (GNB) to predict inverter over-temperature faults due to fan failures, clogged filters, and other related issues. From the tested models, the ANNs achieved the highest performance in predicting the specific faults with a 98.7% accuracy and 98.0% F1-score.

## 1. Introduction

The maritime industry is considered the backbone of international trade, as it is responsible for moving more than 80% of goods around the globe for the sake of trade [1,2,3]. The report on the “Ocean Economy in 2030” by the Organization for Economic Cooperation and Development (OECD) predicts that by 2030, the maritime industries will double their contribution to global value creation, driven by the growing demand for shipping, shipbuilding, marine equipment services, and offshore energy projects [2]. However, these predictions are completely dependent on the integration of technological innovations and developments.

In particular, the significant economic growth before and after the global COVID-19 pandemic crisis, as well as the increase in cargo volumes, have driven maritime ports to develop their capacities in unexpected ways. Shipping lines, as key stakeholders, have consistently pushed for enhanced port performance. On the one hand, shipping alliances, as a means of capacity rationalization, lead to further concentration and to a decreased number of ports to call. Therefore, fewer ports are required to serve more ships. On the other hand, fewer ports are now responsible for accommodating a larger share of global maritime traffic. At the same time, the ongoing increase in vessel size continues to heighten operational pressures on ports. The average size of new container ships delivered has increased from an average of 1k TEUs (Twenty-Feet Equivalent Units) in the 1970s to 7.7 k TEUs ordered today [4]. As bigger ships also mean bigger volumes to be loaded/unloaded within small time windows, this also consequently impacts transferring congestion pressures towards container terminals. Thus, this evolution has provided remarkable needs for the appropriate performance of container handling and logistics. However, operational missing links and bottlenecks remain, including, among others, performance inefficiencies, labor accidents, increased energy consumption, as well as pollutant and Greenhouse gas emissions.

In parallel, the advancement of new digitization paradigms such as cloud-edge computing, internet of things (IoT), big data, and machine learning (ML) has created new possibilities for the industry, leading to the well-known concept of Industry 4.0 [5]. In regard to the cargo handling industry, Industry 4.0 services, also known as Port 4.0, could improve processes by connecting all equipment and systems in real time, thus enabling seamless data exchanges. Under these new conditions, more automated and interoperable solutions could be achieved by the sector with less risk, at a lower cost, and with a faster lead time [6]. Therefore, timely fault detection is very crucial in any industry to avoid long downtime and degradation in performance [7,8].

**Figure 1:**
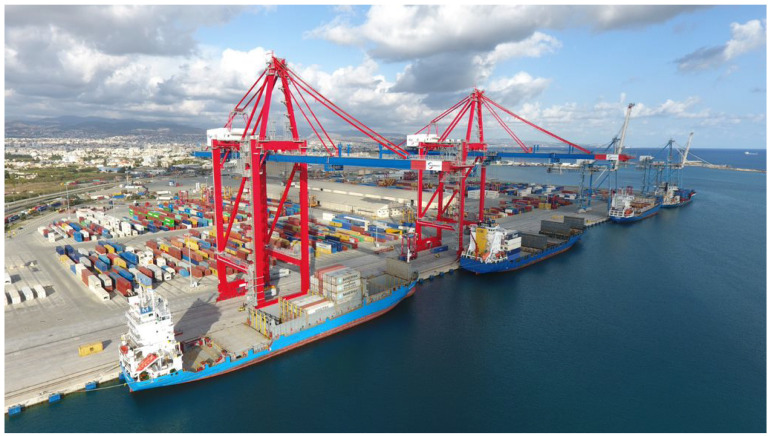
A view of the EUROGATE Container Terminal Limassol (ECTL) at the Port of Limassol, Cyprus, where the operational data used in this study was collected.

In recent decades, due to the high growth in international seaborne trade and the increase in the number of ships, congestion and safety problems have arisen in multiple ports around the world [9]. Figure 1 shows the EUROGATE Container Terminal Limassol (ECTL) at the Port of Limassol, Cyprus, with several cranes and other container handling equipment (CHE). ECTL provided the operational data and the real-world evaluation of the approaches developed in this study. Several decisions and inspections at the port, particularly at container terminals, are still performed manually, such as verifying machine health and inspecting container security seals, which hinders the overall efficiency of terminal operations [10]. Furthermore, unplanned maintenance of port equipment, for example, quay cranes and straddle carriers, also causes a reduction in the performance of container terminals. To avoid such types of problems and to improve the performance of container terminals, it is necessary to adopt the latest technologies for the detection of faults and the fast maintenance of terminal equipment. Intelligent fault detection and predictive maintenance of terminal equipment can help reduce unplanned maintenance activities. For example, predictive maintenance (PdM) results in up-to-date knowledge of the health status, which allows neither waiting for equipment to shut down (i.e., reactive maintenance) nor performing maintenance when it is not required (i.e., preventive maintenance). Normally, CHE such as STS cranes, RTG cranes, straddle carriers, or reach stackers, require an optimal maintenance process due to the heavy loads, long operating times, and diverse weather conditions. Therefore, a precise PdM system is required to achieve the highest performance of the CHE at container terminals.

Preventive maintenance for CHE at the ports has traditionally been performed using SCADA systems set up with human-coded thresholds, warning rules, and configurations to determine when a machine’s condition requires repair or even replacement. However, this semi-manual approach does not take into account the more complex dynamic behavior of the machines, nor the contextual data that relates to the operational process as a whole. For this reason, and thanks to recent advances in AI and IoT, the implementation of ML-based solutions is seen as the next functional step that can lead to significant cost savings, higher predictability, and better availability. Specifically, the training process of ML algorithms enables the detection of anomalies and the testing of correlations when searching for patterns in the various data streams from IoT devices and systems, such as sensors, programmable logic controllers (PLCs), and weather stations. For instance, deep reinforcement learning techniques such as DDPG have been effectively employed for joint time and energy management in intelligent sensors, demonstrating their potential for intelligent resource allocation in complex IoT scenarios [11]. Nowadays, ML-based approaches for PdM are playing an essential role in various domains, i.e., aircraft engine [12,13], smart vehicles [14], energy systems [15,16,17,18], elevator systems [19], and marine engines [3,20,21].

In this work, we propose and test a Machine Learning (ML) framework for predictive maintenance (PdM) operations of container handling equipment (CHE) at smart ports. In particular, the contributions of this study are threefold:

- A comprehensive data collection framework for predictive maintenance applications was designed and deployed for the container terminal at the Port of Limassol, Cyprus. This was achieved by installing multiple sensors on straddle carriers and other CHE and deploying several frameworks to gather real-time operational data. The collected data was then subjected to extensive preprocessing, through cleaning and structuring, to ensure data integrity and usability for predictive maintenance applications.
- Five different ML models were developed and tested for predicting faults related to over-temperature in CHE: artificial neural networks (ANNs), decision trees (DTs), random forest (RF), extreme gradient boosting (XGBoost), and Gaussian Naive Bayes (GNB). Additionally, a statistical model was designed and specifically tailored for anomaly detection in the hydraulic system of straddle carriers.
- Extensive experiments were performed to validate the proposed models, ensuring their effectiveness in real-world predictive maintenance scenarios. The experimental results provided insights into the reliability and accuracy of the different models, demonstrating their capability to detect and predict faults for the considered applications.

The remainder of this paper is organized as follows. Section 2 discloses the literature review, while Section 3 presents the proposed methodology of this study. Section 4 and Section 5 describe the two considered case studies: hydraulic anomalies on straddle carriers (SCs) and inverter over-temperature faults prediction on SCs, respectively. Section 6 discusses key insights from the proposed methodology. Finally, Section 7 concludes the study.

## 2. Literature Review

Predictive maintenance has been applied during the last decades in industrial settings, resulting in a demonstrated improvement in the scheduling of maintenance tasks, the avoidance of downtime, and the improvement of the cost-benefit balance [22,23,24]. These initial techniques have since been refined, and the most recent artificial intelligence (AI) applications and ML algorithms represent a breakthrough solution for obtaining the maximum benefits. While AI- and ML-led models are yet to be applied to terminal yard machinery, previous examples serve to illustrate the results that could be expected from the application of such techniques.

The automobile industry has applied PdM for the maintenance of air pressure systems. In [25], two different classifiers are compared based on ML models, highlighting the potential of these techniques to save money by predicting faults and breakdowns. Shafi et al. develop a PdM method based on ML [14]. They consider the prediction of faults in four subsystems of vehicles, i.e., the ignition system, fuel system, cooling system, and exhaust system. Data is collected when the vehicle is moving under both normal and faulty conditions. Then, the collected data is analyzed, and based on these patterns, PdM is performed for other vehicles. Four classifiers are used: decision tree, SVM, random forest, and nearest neighbor.

The manufacturing industry is also benefiting from the application of ML techniques in the search for decreasing costs and attaining more sustainable operational management [26]. A predictive maintenance system was developed for production lines in manufacturing, which utilized IoT data and machine learning models such as Random Forest and XGBoost to detect potential failures ahead of time [27]. The list of examples goes on, and a deeper systematic review of PdM initiatives in the industry sector can be found in [23,28].

A study presented in [12] also develops a PdM model of an aircraft engine using an autoregressive moving average model. The purpose of this study is to extract useful information from raw sensor signals of an aircraft engine in order to schedule maintenance activities. A total of six data-driven models were implemented and compared in this study, showing that the support vector machine (SVM) achieves higher performance than its counterparts. Another study in [13] addresses PdM related to aircraft fuel systems to reduce maintenance costs and increase flight security. A novel hybrid algorithm (DQINN) that combines a deep belief network and a quantum-inspired neural network to increase the accuracy of the model is developed. Experiments have been conducted on public data, and the results show that the proposed model has higher accuracy than its counterparts.

The work presented in [15] predicts faults in wind turbines by using a Random Forest model. The proposed model consists of three modules: (1) a predictive model for each monitored wind turbine using a random forest algorithm, (2) a monitoring agent that makes predictions every 10 min about failures in wind turbines during the next hour, and finally, (3) a dashboard on which the given predictions can be visualized. The authors of [29] also perform PdM for wind turbines and develop a deep neural network (DNN). The lubricant pressure data is utilized to check the health condition of the wind turbine gearbox. Six other ML models are also implemented for comparison purposes. Based on experiments with real data from wind farms in China, the proposed DNN-based method seems to be more accurate.

In the maritime industry, a systematic approach using big data and ML is proposed to develop predictive maintenance strategies for marine engines [20]. To investigate the proposed model, a case study is conducted on a ship in collaboration with a Norwegian shipping company. The company provided all operational data from the ship’s condition monitoring systems, especially sensor-based information from the engines and compressors. Based on experiments with real data, the proposed method achieves a good accuracy in predicting faults. The studies presented in [21,30] also perform PdM using different ML models to predict faults in the ship engine and other components. The proposed methods are validated by experiments, and the results show the effectiveness of the ML-based methods. Another ML approach has been developed in the shipping industry for the PdM of the electrical propulsion system of ships in [3]. It uses a Balanced Random Forest and multiple instance learning based on event logs of ship electric propulsion systems. The objectives are predicting failure likelihood, time to failure, and explainable predictions to ensure timely crew intervention. The proposed PdM approach is tested through multiple events collected over two years of seagoing ships. From the results, it is observed that the proposed method accurately predicts actual propulsion failures and performs better compared to other methods.

The authors of [31] explore the feasibility of PdM for container-handling cranes by employing traditional ML models (i.e., logistic regression and genetic algorithm), while using simulation-generated synthetic data. Due to limited real-world maintenance data, the study simulates four years of operational data for mobile harbor cranes, focusing on critical components like the hoist motor and gearbox. Various sensors and failure patterns are modeled to generate component health data. The study evaluates prediction accuracy across multiple scenarios with varying failure rates and sensor data complexity. Based on experiments, it is observed that the genetic algorithm showed higher performance than its counterparts. A study presented in [32] also proposes PdM for port handling equipment. The paper emphasizes how AI-driven models can analyze data from maintenance logs to anticipate equipment failures, reduce unplanned downtimes, extend equipment life, and optimize maintenance schedules in port operations. Real data from a Tunisian port is collected and utilized for experiments, and a supervised ML model (K-Nearest Neighbors) was trained to classify forklift status (working/failing) using 13 input features, including equipment specifications and maintenance history. They have a very small amount of data, and the model achieves an average of 64% accuracy using 5-fold cross-validation.

Based on the current literature, a significant amount of work has already been carried out regarding predictive maintenance approaches to solve problems in various industrial environments. However, it is observed that the maritime industry still needs a lot of work to achieve higher performance at the ports, especially at container terminals. This study cannot find any existing methods/approaches in the literature that are specifically developed for fault prediction in port machinery using operational data. Therefore, this study investigates lightweight ML models (ANN, DT, RF, XGBoost, and GNB) for predictive maintenance at container terminals. While advanced deep learning (DL) models could be applicable in this setting, they typically demand substantial computational resources, longer training times, and more extensive hyperparameter tuning, which pose significant challenges for real-time inference and deployment at the edge. Therefore, this study selects the above-mentioned ML models based on computational efficiency, interpretability, and deployment feasibility in resource-constrained edge devices, such as industrial sensors devices and IoT Gateways.

## 3. Proposed Methodology for PdM

The foundation of the proposed predictive maintenance approach, which integrates IoT technologies, big data, and machine learning models, lies in addressing key technical challenges for enhancing the operational efficiency of key port machinery such as CHE. Effective ML implementation relies on the availability of sufficient historical data to capture patterns of past failures, including mechanical properties, average usage rates, and operating conditions. Labeled data reflecting real-world operational conditions is often difficult to obtain due to limited fault occurrences and noise in sensor data. Ensuring robust validation in such imbalanced scenarios is a key challenge, as standard evaluation metrics may not fully reflect the model’s performance in rare-event detection. However, even with comprehensive datasets, the selection, training, and deployment of the most suitable ML model must align with the computational constraints of the infrastructure, ensuring both performance and feasibility. Although cloud computing can support predictive analytics solutions, running these models on a remote server introduces high latencies that can lead to delayed response times, depending on connectivity. Edge technology can be considered instead as a preferable way to optimize the speed and performance of predictive analytics by running ML models locally. This necessitates the use of lightweight models, which in turn may limit the use of complex deep learning architectures. The adopted methodology is presented below.

### 3.1. Data Life Cycle

From the literature, a complete management of the data cycle is provided by a toolset that responds to each of the stages of this cycle. It is a concept of end-to-end data processing by design, so that all processes can be managed and, therefore, bringing added value at every step to offer advanced user-friendly functionalities through well-defined APIs and user interfaces, greatly reducing the complexity of data management, improving the reliability of the information transmitted, as well as the certainty of the decisions taken and therefore the performance and profitability of the operations that take place there. The methodology followed in this article integrates the following data operations, as discussed in [33].

- *Generation*. This phase occurs naturally and organically in any business with a certain degree of digitization, as data generation is continuously growing as more computerized systems, such as new machinery, etc., are added. In the proposed work, operational data is generated by PLCs installed on each SC.
- *Capture*. This stage involves the incorporation of existing data into a data analytics platform through the reading of industrial and computer communication protocols. In case the equipment is not able to transfer the data, specific equipment that helps to capture the information is integrated. In this study, data capture is conducted via Node-RED custom flows running on wired IoT gateways installed on each SC. These flows acquire data directly from the PLCs and sensors and transmit it using the MQTT protocol to an edge server for further processing.
- *Processing*. In this stage of the data life cycle, several preprocessing steps are applied to ensure data quality and model robustness in real-world industrial environments. Data cleaning is performed to remove duplicates, eliminate empty or invalid fields, fill missing values, and synchronize timestamps. Additionally, calibration is conducted to convert raw machine data, often in binary or unitless formats from PLCs, into meaningful physical units. These steps also help mitigate the effects of sensor degradation and inconsistent sensor calibration.
- *Storage*. The processed information is saved in a reliable, accessible, and scalable system so that it can be used by the rest of the data life cycle components, while guaranteeing that the data follows FAIR principles. In this study, the time-series data is stored in InfluxDB. In parallel, machine health records, fault logs, and maintenance activities are recorded and stored in LIMBLE, a private cloud-based computerized maintenance management system (CMMS) used at the EUROGATE terminal.
- *Analysis*. At this stage, advanced processing operations that allow additional information to be extracted from the stored data are performed. This includes calculation of aggregations, inference of statistics, determination of meaningful data blocks, and association of inferred data with blocks. In this work, both InfluxDB and LIMBLE serve as data sources for further analytics, including the development and execution of machine learning models for predictive maintenance. Analysis includes statistical aggregations, event detection, correlation inference, and fault classification.
- *Visualization*. Data visualization helps to present results in a clear and simple way that a human can easily understand and interpret. In many cases, a graph can represent tens of gigabytes of information in a single image. It is at this stage of the data life cycle that aesthetics needs to be considered, along with functionality and human visual perception, to convey the results of data analysis.

### 3.2. AI/ML Models Lifecycle

Understanding the life cycle of ML models is crucial for developing effective applications for the port environment. Following the ML life cycle outlined in [34], we identify and describe the key phases relevant to our study.

- *Problem Definition and Data acquisition/Collection:* Port experts must define clear objectives based on known outputs. Data acquisition focuses on collecting data relevant to the determined goals.
- *Data Preprocessing:*
  – Data Exploration: Understanding port-related data distributions and relationships between variables, as well as any possible bias inherent to the data life-cycle.
  – Data Cleaning: Remove noise and correct errors to improve data quality.
  – Feature Engineering: Transform raw data into features that enhance predictive power.
  – Data Splitting: Divide data into training, validation, and test sets for unbiased evaluation.
- *Model Selection and Training:* Choose appropriate classification or regression algorithms for the port challenges. Training involves hyperparameter tuning and validation to ensure robustness, often through cross-validation techniques.
- *Model Evaluation:* Use performance metrics such as accuracy, precision, recall, and F1-score to assess the expected model associated with the container terminal needs.
- *Deployment:* Integrate the model into existing systems while considering scalability and efficiency. Implement feedback mechanisms to refine the model post-deployment.
- *Monitoring and Maintenance:* Monitor model performance continuously and address model drift by retraining when necessary. Overcome operational challenges to maintain the effectiveness of ML systems.

While the above outline focuses mostly on supervised models, for unsupervised models, there are a few key changes in the approach entirely. In unsupervised learning, models focus on finding patterns or structures in unlabeled data. Key changes include the following:

- *Problem Definition:* Objectives often center around discovering inherent data structures.
- *Model Selection:* Involves choosing algorithms like clustering, autoencoders, statistical methods, or dimensionality reduction.
- *Evaluation:* Relies on metrics such as silhouette scores, cluster validity indices, or qualitative assessments.
- *Monitoring:* Emphasizes detecting shifts in data patterns rather than predictive accuracy.

### 3.3. Predictive Maintenance Architecture

**Figure 2:**
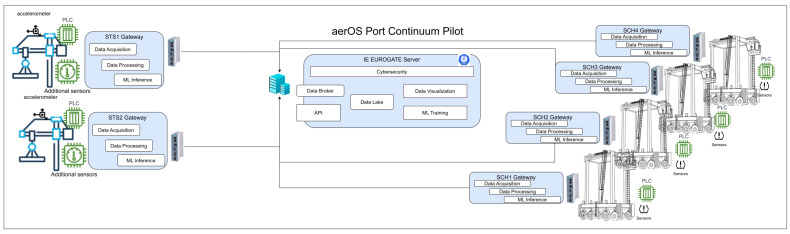
Port continuum architecture for predictive maintenance.

The proposed architecture for the implementation and testing of ML-based predictive maintenance models over CHEs at the EUROGATE Container Terminal Limassol (ECTL), following the data and ML life cycles described above, is presented in Figure 2. The ECTL is responsible for supporting more than 90% of trade in Cyprus, reaching approximately 500,000 TEUs per year. The ECTL is equipped with four quay cranes and 36 straddle carriers. The quay length and depth are 800 m and 16 m, respectively. While considering the ECTL, the primary objective of this study is to deploy various ML algorithms on historical data to detect (1) anomalies and (2) faults in straddle carriers (SCs). An efficient prediction of faults in SCs will help to enhance their performance and reduce the downtime of machines. The SCs are utilized in container terminals to perform the horizontal movements within the port. As can be seen from Figure 2, the generation, capture, and processing are performed in the far-edge gateways that collect data from sensors and STS/straddle PLCs. The processed information is transmitted to the next iteration block in the data life cycle, i.e., data lake or storage, in which the information is retrieved by the analysis component that embeds the algorithms and ML models used for refining the maintenance issues that it was keen on to be predicted in advance of any failure occurring. This architecture and use cases of the current study align with the cloud-edge-IoT continuum concept, ensuring intelligent processing is distributed efficiently across the edge and cloud layers, and is part of the aerOS EU Project [35]. The two case studies were selected to ensure representativeness and generalizability of the proposed predictive maintenance framework. Hydraulic anomalies are often subtle, gradual, and lack labeled data, making them suitable for unsupervised techniques. In contrast, overtemperature faults are usually more abrupt and have clear failure signatures, which allowed us to apply supervised learning. This contrast helps demonstrate the flexibility of the framework across varying fault types and learning paradigms.

In this study, four electric SCs are monitored, where each SC is equipped with PLCs (Omron model CS1G-CPU44H, Japan) featuring a Pn/DP CPU, 1.5 MB of work memory for programs, and 5 MB for data storage. These PLCs generate operational time-series data. In addition, hydraulic pressure sensors have been installed onboard, providing additional contextual data for the case study explained in Section 4. The hydraulic sensors installed are Danfoss MBS 3000 Pressure transmitters (Nordborg, Denmark) rated for an accuracy of ≤±0.5% typical, (≤±1% max) (both full scale). The SCs are also equipped with IMUF99 inclination and acceleration sensors (Mannheim, Germany), VIM32PL vibration sensors (Mannheim, Germany), a Kinco Human Machine Interface (HMI) (Shenzen, China), and a Teltonika RUT240 M2M/IoT 4G router (Vilnius, Lithuania). All this information is captured by means of low-code Node-Red [36] custom flows mounted on top of wired IoT gateways located in the SCs. Next, these signals are transmitted to an edge server using the MQTT protocol [37], which is further collected on different data lakes for analysis, characterization, and inference. Furthermore, InfluxDB [38], an open-source time series database, is employed to store all the data. Additionally, machine health, fault records, and maintenance activities are documented in LIMBLE, a private data management system used at the EUROGATE container terminal. This system collects data both directly from PLCs and through manual input by technicians at the terminal. So, in conclusion, this study exploits data from two platforms, LIMBLE and InfluxDB, for further experiments and investigation.

## 4. Case Study 1: Hydraulic Anomalies on Straddle Carriers

Most mechanical actuators inside straddle carriers are hydraulic, including the parking brake, steering, and spreader. For this reason, monitoring and predicting the status and health of the hydraulic system are crucial for the operation of the machine.

### 4.1. Background

The hydraulic system of every industrial system utilizes pumps that work in bursts to maintain the system pressure over a given threshold. Under the usual operation of the SCs, the hydraulic pump activates when the system pressure is under a lower threshold and runs continuously until an upper threshold is reached. This creates a roughly inverted sawtooth profile in the pressure-time curve with a period of around 60 s when the SC is idle. Naturally, the frequency of those peaks is highly dependent on the behavior of the crane at a given moment. That is, some tasks require a higher pressure demand (such as operating the spreader) and cause the pump to fire more frequently.

Some events, such as a leak or a clogged fitting, can have a noticeable impact on the pressure of the whole system or the duty cycle of the pump, e.g., requesting pressure continuously to replace the air lost due to the leak. Thus, by monitoring the duty cycle of the pump (which under normal operation only fires in pulses), some of those issues can be detected before causing significant damage.

### 4.2. Methodology

The anomaly detection is carried out using a custom variation in the usual Kolmogorov–Smirnov test using the 95th percentile (the value 95 was selected experimentally as the highest value that provided satisfactory robustness) instead of the supremum for increased robustness in the presence of extreme values in long-tailed distributions. This approach, based on traditional statistical methods, was deemed more suitable due to the lack of appropriate labels to train a supervised ML model. Instead, pressure signals are compared against signals from periods where machine operation is verifiably correct (behavior characterization).

**Figure 3:**
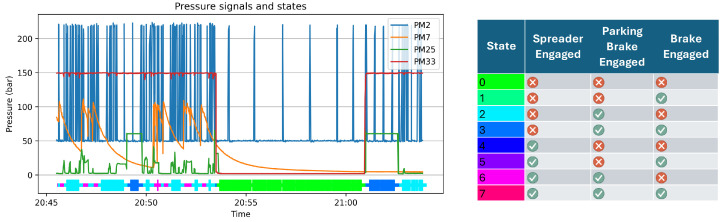
Plot of the pressure measurement at the pump’s output (PM2), the three hydraulic signals used to determine the state of the crane (PM7, PM25, and PM33), and the identified state in the color bar below. On the right is a table detailing the specific combination of the three signals that define each of the eight states.

After a simple clean-up and formatting of the data, each observation is binned in different states according to which subset of the hydraulic actuators is in use. The three actuators of interest are the pressure measurement (PM) for the spreader (PM7), the parking brake (PM33), and the service brakes (PM25). Binary combinations of these actuators being pressurized or not result in eight different states. Although some of those states do not occur (or rarely do so), such as those involving using the service brakes while the parking brake is engaged (states 1 and 5), only states 0, 2, 3, and 6 occur regularly (Figure 3).

**Figure 4:**
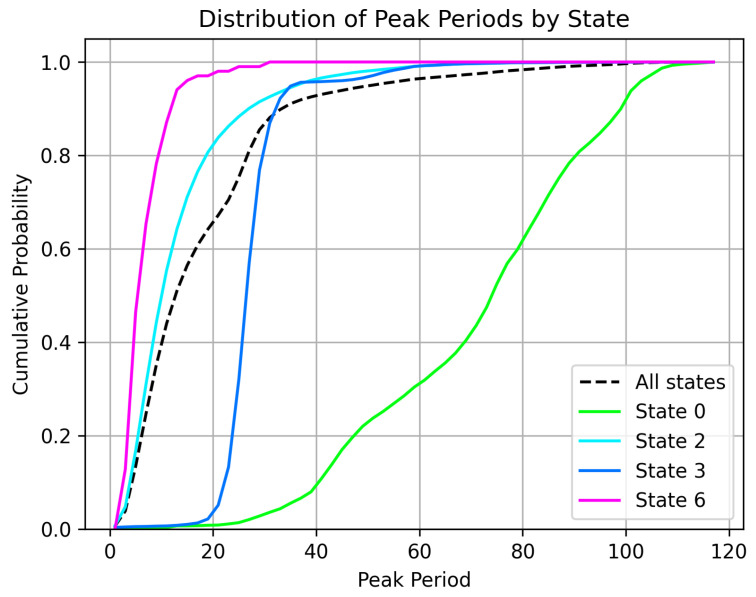
Cumulative distribution of the period between consecutive peaks under regular operating conditions segregated by states. Only common states are shown.

By segregating the behavior into these states, we can expect the duty cycle of the pump to be rather homogeneous in terms of pressure demand. This way, any relevant deviations are indicative of anomalies in the system. These deviations can be observed by comparing a few key statistics of the hydraulic signals against a reference distribution computed for each state from the periods of time where the crane is guaranteed to be in working order. For this study, we have focused on the period between consecutive peaks. Figure 4 illustrates how the distribution for the period between consecutive peaks of the pressure measurement at the pump’s output (PM2) is quite different for different states (behaviors) and fairly consistent within each state.

This comparison is performed by computing the residuals between the quantiles of reference and those of the distribution in the most recent hour for each of the states and considering different quantiles of the absolute value of the residuals. The two estimators used to flag anomalies are computed as $q_{0.1}$ and $q_{0.9}$ from the distribution of

$$\left\{|F_{ref}\left(x\right)-F_{obs}\left(x\right)|:x\in \left[0,1\right]\right\}$$

**Figure 5:**
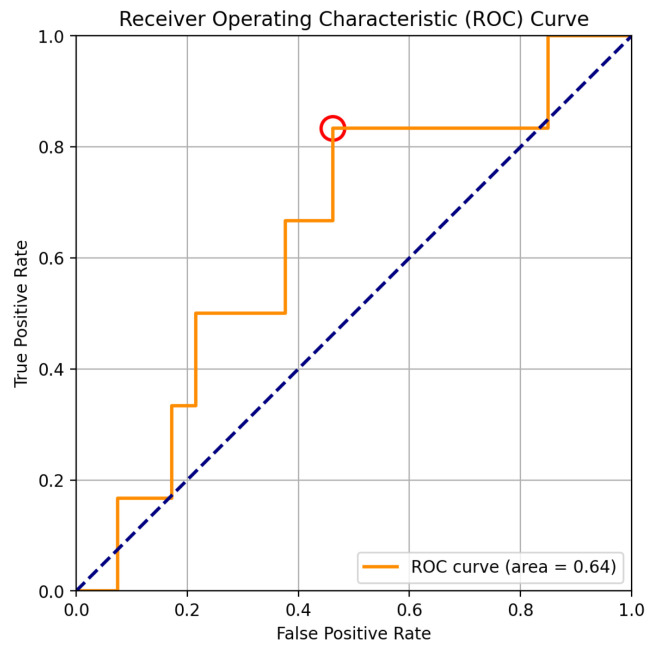
Receiver Operating Characteristic (ROC) curve with parameter $q_{0.9}$ illustrating the trade-off between the True Positive Rate and the False Positive Rate for the anomaly detection model. The red marker highlights the selected threshold that optimizes detection balance. The dashed blue line serves as a baseline for random classification.

Higher values of either of these estimators indicate higher deviations in the observed distribution for the most recent period as compared to the reference distribution under regular operation. However, given the reduced size of the hand-labeled dataset and its unbalanced nature, a decision rule based exclusively on $q_{0.9}$ was chosen to avoid overfitting the criterion. Thus, an observation is considered to be anomalous if $q_{0.9}$ is greater than some threshold determined with the aid of the ROC curve pictured in Figure 5. The threshold value 1.5 (marked with a red circle in the figure) provides the results shown in Section 4.3, which was found to provide the best (lower) False Positive Rate (precision) without compromising True positive rate (1—sensibility) which is considered crucial in order to miss as few true positives as possible.

### 4.3. Experimental Setting and Results

Faced with the lack of high-quality labels, we employed an unsupervised anomaly detection method based on more traditional statistical tests to identify potential anomalies in the dataset, which consists of time series for the four hydraulic signals measured every 100 ms from June to October 2024, totaling 4 GB. This approach generated a list of potentially anomalous timestamps, each assigned a likelihood score indicating their rarity as anomalies. Statistical analysis validated the significance of the detected outliers. To further ensure the quality of the developed method, three main metrics were considered (precision, recall, and accuracy). A domain expert manually reviewed a subset of flagged timestamps over a continuous 5-month period (June–October 2024).

As can be seen in the Confusion Matrix on the left part of Table 1, during that time frame, 499 timestamps were considered, of which, in turn, 45 of them were flagged as potentially anomalous by the model. In parallel, the engineering team informed about six actual anomalous events. By comparison of these two reported labels, it was determined that 83% of the actual anomalies were correctly flagged as potential anomalies by the model. In the right part of Table 1, the binary metrics lead to a model with low recall due to a large quantity of false positives but a high precision. In that sense, for this particular case study, the most relevant metric is precision since the cost of a false negative is significantly higher than that of a false positive. Other relevant metrics are accuracy with 91.8% and specificity (True Negative Rate) with 99.78%.

These results demonstrate that our unsupervised technique effectively identifies meaningful anomalies even in the absence of labeled training data.

## 5. Case Study 2: Detecting Overtemperature Faults in Straddle Carriers

This section discloses the case study related to detecting overtemperature faults in straddle carriers. Then, the section unfolds the developed models along with the simulation settings and results.

### 5.1. Background

In this second case study, we focus on predicting inverter overtemperature faults in SCs, a critical fault that occurred six times during the year 2024. These faults directly affect the cooling system of the SCs, leading to elevated inverter or engine temperatures. If left unaddressed, this issue can escalate, potentially resulting in a complete shutdown of the engine or the entire straddle carrier, thereby causing operational delays and safety risks. To develop and evaluate our ML models for inverter overtemperature fault prediction, we selected four of the six recorded failure incidents for training purposes, while the remaining two incidents were reserved for testing. This setup enables the model to learn from historical failure patterns and assess its ability to generalize to unseen events.

### 5.2. Methodology

The analysis is based on real-world telemetry data collected from 15 SCs operating at the EUROGATE Container Terminal Limassol. The data originates from the straddle carriers’ PLC and other installed sensors, such as hydraulic pressure, inclination, acceleration, and vibration sensors. Over 200 measurements are recorded every few minutes of operation per straddle carrier, including inverter, motor, and engine temperatures, speed, torque, hydraulic pressure, and various error flags. Ambient temperature readings were also recorded using an on-site weather station and incorporated into the dataset. The dataset was manually labeled to consist of two classes: normal and faulty. Faulty data were identified from SCs involved in the recorded incidents, while data from other SCs operating simultaneously were labeled as normal. In total, six inverter overtemperature fault incidents were identified between January and December 2024. The four incidents were used for training, and the two for testing to ensure that the models are tested with incidents they had never observed before. The training dataset size is 8.1 MB and contains 20,210 records (13,860 normal and 6350 faulty), while the testing dataset size is 2.1 MB and includes 5192 records (3947 normal and 1245 faulty). During data preprocessing, missing values were filled using the mean of each metric, one-hot encoding was used for categorical variables, and all numerical variables were standardized by removing the mean and scaling to unit variance.

To perform predictive maintenance utilizing historical data, five popular machine learning models have been evaluated.

1. *Artificial Neural Network (ANN):* The ANN is capable of capturing complex and non-linear relationships in the data. It consists of layers of neurons, where each neuron applies a transformation to the input and passes it to the next layer.
2. *Decision Tree (DT):* The decision tree classifier is a rule-based model that splits the dataset based on feature values to form a tree-like structure of decisions.
3. *Random Forest (RF):* RF is an ensemble learning technique that constructs multiple decision trees and combines their outputs to improve predictive accuracy and reduce overfitting. Each tree is trained on a random subset of data and features, enhancing model robustness.
4. *Extreme Gradient Boosting (XGBoost):* XGBoost is a powerful, optimized gradient boosting algorithm that builds an ensemble of decision trees sequentially to minimize prediction errors. It utilizes regularization techniques to improve generalization and reduce overfitting.
5. *Gaussian Naive Bayes (GNB):* GNB is a probabilistic classifier based on Bayes’ theorem that assumes features are conditionally independent given the class and that each feature follows a Gaussian (normal) distribution.

Feature importance was determined by training each model and computing feature importance using the recommended approach for each model. Specifically, we computed permutation importance for ANN, impurity-based importance for DT, RF, and XGBoost, and the log of the coefficients for GNB. Next, we calculated the average importance of each input variable and kept all features with a non-zero importance. In addition, SHAP (SHapley Additive exPlanations) analysis [39] was applied to all models to interpret the contribution of each feature to the model’s predictions. Finally, the model hyperparameters were optimized using a 5-fold cross-validated grid search over a parameter grid. The performance of the models for fault prediction was evaluated using accuracy, precision, recall, and F1-score.

### 5.3. Experimental Setting and Results

This study uses the data from the last year (January 2024 to December 2024) to train and test ML models for predicting inverter overtemperature faults. The PdM models are developed in Python v3.12.2 using the scikit-learn (v1.5.2) framework. The experiments were conducted on a computer system equipped with an AMD EPYC 7352 24-core processor (2.3 GHz), 64 GB of RAM, and running Ubuntu 22.04.4 LTS.

**Figure 6:**
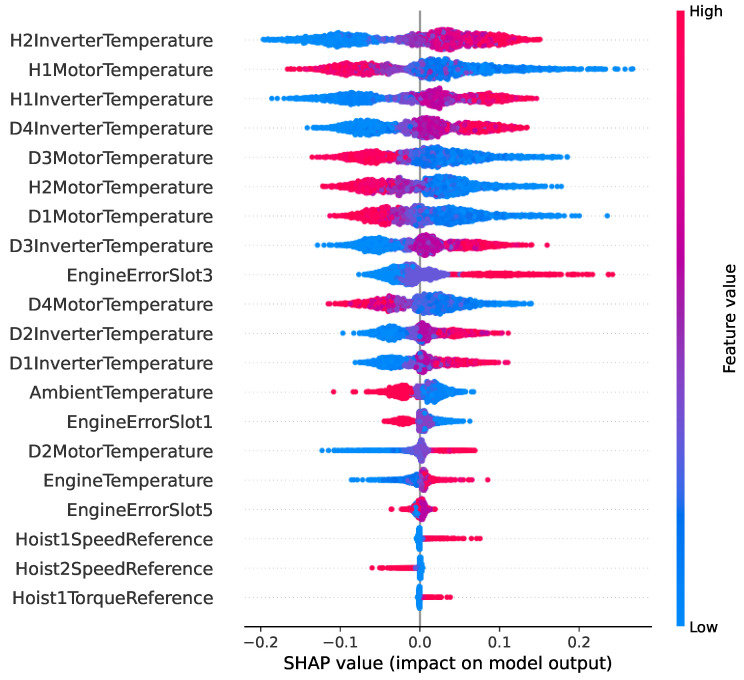
SHAP violin summary plot showing the distribution of feature contributions to the PdM model’s predictions.

Table 2 lists the 21 selected features along with the computed average feature importance for the inverter overtemperature fault prediction. The most important features include the hoist (H) and drive (D) inverter and motor temperatures, which are directly impacted by this type of inverter failure. The AmbientTemperature is also a good feature to have as it helps the model differentiate cases of increased inverter temperatures due to weather conditions as opposed to an inverter fault. To further investigate the impact of each input feature on the output of our predictive maintenance model, we employ the SHAP analysis and visualize the results from the ANN model in the SHAP violin plot in Figure 6. The x-axis shows the SHAP value, which quantifies how much each feature contributes to increasing or decreasing the predicted risk of failure. Positive SHAP values indicate a push toward predicting failure, while negative values suggest a reduced risk. The y-axis lists the features, ranked from top (most impactful) to bottom (least impactful). The color gradient represents the actual value of the feature, with blue indicating low values and red representing high values.

The SHAP violin plot in Figure 6 reveals that the model relies heavily on motor and inverter temperature features, with H2InverterTemperature, H1MotorTemperature, and H3InverterTemperature contributing the most to predictions (ranked high in Table 2 as well). The wide spread in SHAP values, especially for these features, suggests strong nonlinear relationships with the target variable. These variables significantly influence the decision boundary and are critical for fault detection or condition classification in this domain. Other features like AmbientTemperature and EngineErrorSlot3 show moderate influence, while torque and speed references appear to have a smaller impact. These insights help highlight which operational conditions most affect the model’s predictions.

Hyperparameter tuning was performed using grid search and a 5-fold cross-validation. Table 3 lists the parameter space used for each ML model as well as the optimal parameter value that yielded the lowest F1-score. For each hyperparameter, a range of candidate values was determined based on the existing literature and the model-specific documentation [40,41,42].

**Figure 7:**
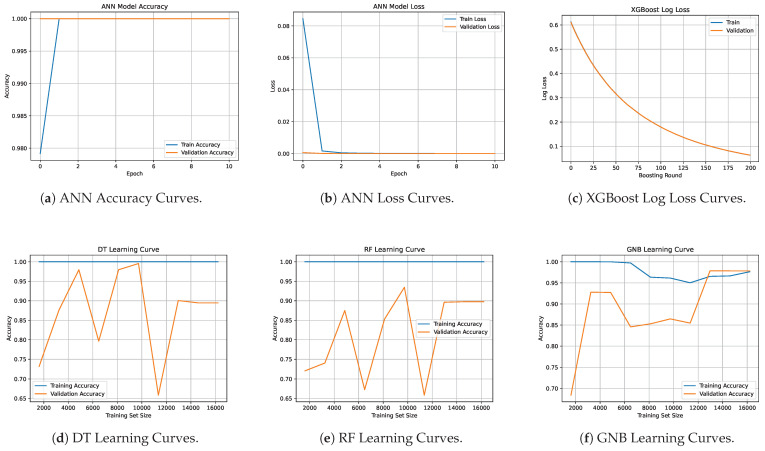
Training and validation learning curves for the five ML models.

Learning curves provide a valuable diagnostic tool for evaluating model convergence and generalization behavior as a function of training set size or number of training rounds/epochs. In this work, we generated learning curves, shown in Figure 7, for all models to gain insights into their learning dynamics and capacity. As ANNs are the only models trained over multiple epochs, we plotted epoch-wise training and validation accuracy and loss (see Figure 7a,b) to monitor convergence, potential overfitting, and the effectiveness of early stopping strategies. Interestingly, both training and validation loss curves rapidly converged to near zero within just three epochs, indicating that the model was able to fit the data early during training and that the network had sufficient capacity to learn it without risk of underfitting. For the XGBoost model that is trained in boosting iterations, we plotted log loss across boosting rounds (see Figure 7c). The training and validation loss curves for XGBoost exhibited a smooth and consistent downward trend across boosting rounds. Notably, the two curves closely overlapped, indicating strong generalization and minimal overfitting throughout training. Finally, for the non-iterative models, i.e., DT, RF, and GNB, we constructed learning curves showing accuracy on both training and validation data as a function of training set size. The learning curves for the DT (see Figure 7d) and RF (see Figure 7e) show perfect training accuracy across the training set sizes, indicating overfitting. In contrast, the validation accuracy fluctuates at the beginning, reflecting sensitivity to training samples, but stabilizes and plateaus around 90%, suggesting improved generalization with more data. Finally, the GNB learning curve (see Figure 7f) shows a gradual decline in training accuracy as the dataset size increases, indicating reduced overfitting. The validation accuracy improves correspondingly and eventually converges with the training accuracy around 97%, suggesting good generalization performance and a well-balanced bias-variance trade-off.

**Figure 8:**
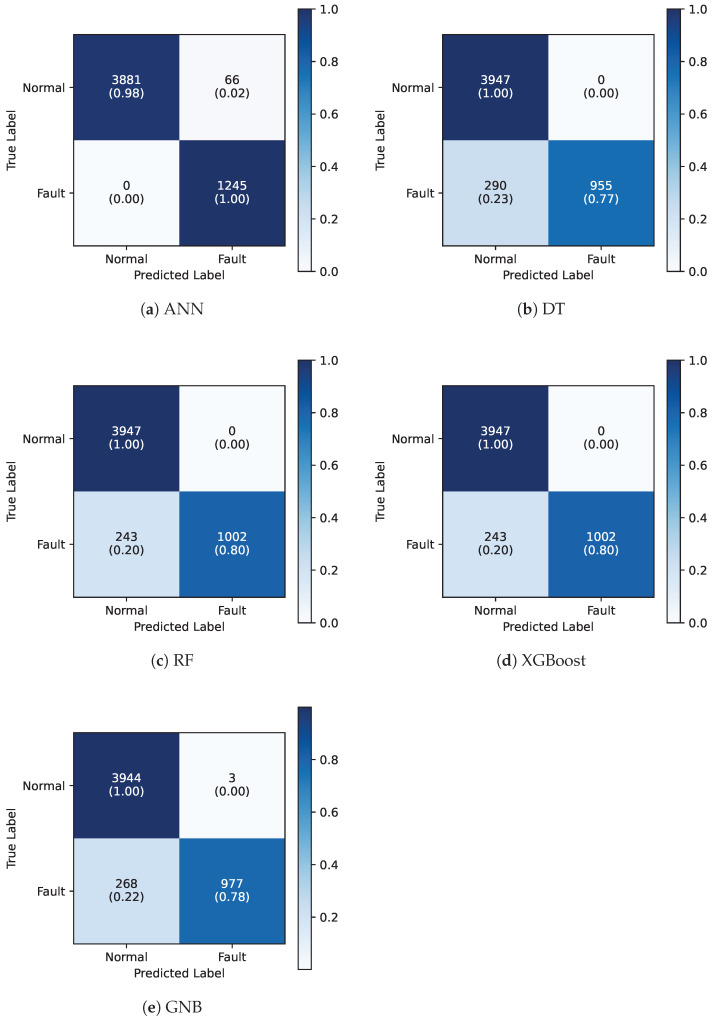
Confusion matrices for the five ML models.

Table 4 shows the performance results of the five ML models on the test dataset using four common classification performance metrics, namely, accuracy, precision, recall, and F1-score, while Figure 8 shows the corresponding confusion matrices. The ANN model outperforms others across all metrics with a 98.7% accuracy and 98.0% F1-score, likely due to its superior capability in capturing nonlinear relationships between input sensor variables and the target fault condition, as well as due to its multilayered architecture and ability to learn complex feature representations. The ensemble-based models, such as Random Forest and XGBoost, demonstrated identical performance with an accuracy of 95.4% and an F1-score of 97.0%. Interestingly, the ANN model did not classify any of the normal data points as faulty, with only a small number of false positives (2%), while RF and XGBoost achieved zero false positives (see Figure 8). These results suggest that these models are highly effective at capturing fault patterns and minimizing misclassification. Further to ensemble-based models, GNB showed a lower performance with an accuracy of 94.8% and F1-score of 92.5%, likely due to its strong assumption of independence between features. Finally, even though DT also did not produce any false positives, DT achieved the lowest accuracy at 94.4% and an even lower F1-score of 91.5% because of its increased false negative rate. Overall, these results indicate that the ANN model effectively captures temporal dependencies within the data, making it highly suitable for predictive maintenance tasks.

## 6. Discussion

Although the current study provides solutions for PdM by developing ML models, it can also offer transformative benefits to container terminals and ports, acting as a cornerstone for operational efficiency and enhanced asset reliability. By leveraging IoT, AI/ML, and advanced analytics, these systems enable early detection of equipment anomalies and potential failures, thus minimizing unexpected downtime and ensuring seamless port operations. In PdM-based systems, the generation, capture, and processing are performed in the far-edge gateways that collect data from sensors and straddle PLCs. Then, the processed information is transmitted to the next iteration block in the data life cycle, in which the information is retrieved by the analysis component that embeds the algorithms and ML models used for predicting in advance of any failure occurring. This approach not only extends the lifespan of critical machinery such as cranes, forklifts, and conveyor belts but also optimizes maintenance scheduling, reducing labor costs and preventing costly emergency repairs. Furthermore, this study can help policymakers and terminal managers in several decisions, i.e., maintenance scheduling, optimized spare-parts inventory, safety and risk management, and minimized operational delays.

In that sense, current preventive maintenance tools, like the ones used at the EUROGATE Container Terminal Limassol, plan the maintenance tasks according to some number of working hours. This sub-optimal approach is frequently not enough for removing any unexpected failure of CHE components, and undesired idle times at operational hours occur. With a total downtime estimated in 2023 of 900 h, the proposed ML models are expected to provide a significant reduction in CHE idle time due to failures. Finally, by implementing this study at the port, the container terminal will gain business value in terms of cost reduction, with minimum delays and equipment downtime due to unexpected failures, achieving sustainability, increased throughput, and gaining a strong reputation (attracting more shipping lines).

While developing predictive models for container terminals, this study encountered several challenges, primarily due to the lack of real-world data, particularly faulty records from the Port of Limassol. In many cases, terminal operators face challenges in sharing their data, making it difficult to validate the developed models across different environments and ports. A significant limitation of this study is the scarcity of faulty records and the high effort required to manually label historical telemetry data based on recorded failure incidents. Additionally, reliance on sensor data poses a challenge in building an autonomous fault detection system, as data quality and consistency can impact model accuracy. Lastly, while the trained models demonstrate promising results under specific terminal conditions, they may require retraining to adapt to different environments with unique operational factors.

## 7. Conclusions

This study investigates the use of different AI/ML on a predictive maintenance service, paramount for the efficiency and safety of container terminals. The data is collected from the EUROGATE Container Terminal at the Port of Limassol in Cyprus. Both faulty and normal operational data have been collected during two years for further training and validation of the developed ML models.

On the one hand, a traditional unsupervised model for the hydraulic system of straddle carriers has been implemented due to the lack of appropriate labels. The proposed model achieves a precision of 83.3%, which is considered the most relevant metric given the disproportionate cost of false negatives (an anomaly being undetected) over false positives.

Furthermore, five different models, including ANN, DT, RF, XGBoost, and GNB, have been developed using real-world historical data to detect inverter overtemperature faults due to fan failures, clogged filters, and other related issues. The results show that the ANN achieves a higher accuracy of 98.7% in predicting anomalies, closely followed by RF and XGBoost with the same value of 95.3%. On the other hand, DT and GNB are not performing well and achieve an accuracy of around 94% in predicting faults.

As future work, further testing is required to validate and enhance the approach for the hydraulic anomaly detection model, since the utilized dataset contained a small number of anomalies. Expanding this dataset or applying the model to similar scenarios will be carried out in the future. Furthermore, we plan to extend the ML-based predictive maintenance tasks to other types of faults, such as engine, brake, and other inverter issues.

## Figures and Tables

**Table 1:** Confusion Matrix and classification metrics for the hydraulic anomaly detection model.

Confusion Matrix				Binary Classification Metrics	
Predicted				Metric	Value
**Actual**	Positive	Negative	**Total**	Accuracy	0.918
Positive	5	1	6	Precision	0.833
Negative	40	453	493	Recall (Sensitivity)	0.111
**Total**	45	454	499	Specificity	0.998

**Table 2:** Feature importance for inverter overtemperature fault prediction.

Feature	Average	Feature	Average
H1InverterTemperature	0.612	D4InverterTemperature	0.184
D1MotorTemperature	0.468	Hoist2SpeedReference	0.179
D2InverterTemperature	0.448	Hoist1SpeedReference	0.179
H2InverterTemperature	0.341	D3MotorTemperature	0.179
D3InverterTemperature	0.229	H2MotorTemperature	0.176
H1MotorTemperature	0.203	D2MotorTemperature	0.158
Hoist2TorqueReference	0.200	EngineErrorSlot1	0.158
AmbientTemperature	0.198	EngineErrorSlot5	0.154
D1InverterTemperature	0.195	EngineTemperature	0.144
Hoist1TorqueReference	0.194	D4MotorTemperature	0.136
EngineErrorSlot3	0.193		

**Table 3:** Hyperparameter tuning parameter space and optimal values for each ML model.

Model	Parameter	Options	Optimal
ANN	batch_size	16, 32, 64	64
	epochs	10, 20, 50	20
	model_dropout_rate	0.0, 0.2, 0.3, 0.5	0.0
	model_neurons	32, 64, 128	128
	model_optimizer	adam, sgd	adam
	validation_split	0.1, 0.2	0.1
DT	ccp_alpha	0.1, 0.01, 0.001	0.001
	criterion	gini, entropy	gini
	max_depth	None, 10, 20, 30	None
	min_samples_leaf	1, 5, 10	1
	min_samples_split	2, 10, 20	2
	splitter	best, random	random
RF	criterion	gini, entropy	entropy
	max_depth	None, 10, 20, 30	None
	max_features	10, 20, 30, 40	10
	min_samples_leaf	1, 5, 10	1
	min_samples_split	2, 10, 20	2
	n_estimators	100, 200, 300	100
XGBoost	colsample_bytree	0.6, 0.8, 1.0	0.6
	learning_rate	0.01, 0.1, 0.2	0.1
	max_depth	3, 6, 9	3
	n_estimators	100, 200, 300	300
	subsample	0.6, 0.8, 1.0	0.6
GNB	var_smoothing	$10^{-9},10^{-8},...,10^{-1},1.0$	1.0

**Table 4:** Performance analysis in terms of accuracy, precision, recall, and F1-score.

Model	Accuracy	Precision	Recall	F1-Score
ANN	0.9873	0.975	0.990	0.980
DT	0.9441	0.965	0.885	0.915
RF	0.9532	0.970	0.900	0.930
XGBoost	0.9532	0.970	0.900	0.930
GNB	0.9478	0.970	0.890	0.925

## Data Availability

The data can be provided on request to the corresponding author.

## Abbreviations

The following abbreviations are used in this manuscript:

AI	Artificial Intelligence
ANN	Artificial Neural Network
CHE	Container Handling Equipment
DT	Decision Tree
DL	Deep Learning
ECTL	EUROGATE Container Terminal Limassol
GNB	Gaussian Naive Bayes
LR	Logistic Regression
ML	Machine Learning
PdM	Predictive Maintenance
PLC	Programmable Logic Unit
RF	Random Forest
RUL	Remaining Useful Life
SC	Straddle Carrier
SHAP	SHapley Additive exPlanations
SVM	support Vector Machine
XGBoost	Extreme Gradient Boosting
